# Predictive role of cystatin C and increased proteinuria in early assessment of acute renal toxicity in patient poisoned by nephrotoxic drugs and poisons

**DOI:** 10.1186/s40360-025-00935-x

**Published:** 2025-05-13

**Authors:** Esam Mohammed Abdallah Ali, Emad Ahmad Mohamad Yousef, Maha Abd El-Hamed Helal, Mohammed Hamdi Mohammed, Meray Medhat Shokry Zaghary, Marwa Ahmed Hasb Elnabi

**Affiliations:** 1 Forensic Medicine & Clinical Toxicology Department, Faculty of Medicine, Sohag University, Sohag, 82524 Egypt; 2 Internal Medicine Department, Faculty of Medicine, Sohag University Sohag, Sohag, 82524 Egypt

**Keywords:** Acute kidney injury, Cystatin C, Proteinuria, Nephrotoxicity, Acute poisoning

## Abstract

**Background:**

Acute kidney injury (AKI) is prevalent in critical care, often due to nephrotoxic drug exposure, which accounts for significant morbidity and mortality. Current biomarkers, like serum creatinine, lack sensitivity for early detection of nephrotoxicity.

**Aim:**

This study evaluates proteinuria and serum cystatin C as early indicators of nephrotoxicity in acutely poisoned patients at Sohag University Hospitals.

**Methods:**

This prospective study involved 100 acutely poisoned patients with nephrotoxic effects admitted to Sohag University Hospitals from April to August 2021. Inclusion criteria required symptomatic patients who provided at least four blood or urine samples, including one within 24 h post-ingestion. AKI was classified using the Acute Kidney Injury Network (AKIN) criteria, with baseline serum creatinine estimated from the lowest value during hospitalization. Biomarkers, including serum creatinine and cystatin C, were measured using standard assays for analysis.

**Results:**

The study included 100 patients aged 2 to 58 years, predominantly male (72%). Most participants were from rural areas (82%). Serum creatinine levels significantly increased from day 1 (mean ± SD: 1.67 ± 0.6 mg/dL) to day 2 (mean ± SD: 2.98 ± 1.35 mg/dL). Significant predictors of acute renal toxicity included serum creatinine on both days (*P* < 0.001), proteinuria ACR (*P* = 0.023), and cystatin C (*P* < 0.001). Cystatin C had the highest predictive value (AUC = 0.993), while proteinuria ACR and day 2 serum creatinine showed significant predictive capabilities (AUCs of 0.805 and 0.873, respectively).

**Conclusion:**

In conclusion, proteinuria and cystatin C are reliable predictors for early nephrotoxicity detection in acutely poisoned patients at Sohag University Hospitals. These biomarkers effectively indicate and assess the severity of kidney injury caused by toxicity.

## Introduction

Acute kidney injury (AKI) is a prevalent condition both in hospital and in pre-hospital situations. Affecting up to 60% of patients admitted to intensive care units. Over recent decades, acute kidney injury incidence has grown, driven by an aging population and increasing rates of chronic kidney disease (CKD) and diabetes mellitus. Nephrotoxicity, the third leading cause of AKI, has become more significant due to the regular use of medicines that may harm the kidneys. Studies show that up to 20% of critical patients are exposed to nephrotoxic drugs [1].

Although new medications undergo safety testing, drug-induced kidney damage often becomes apparent only after market release, particularly as the kidneys’ excretory role exposes sensitive structures, such as the glomeruli and tubules, to high concentrations of exogenous substances [2].

Nephrotoxicity caused by drugs is described as any kidney injury induced directly or indirectly by drugs. It may manifest as nephrotic syndrome, a decreased glomerular filtration rate (GFR), or electrolyte imbalances due to damage to glomeruli or tubules [2]. Epidemiologically, up to 25% of AKI cases are attributed to drug toxicity, with 20% of these patients requiring renal replacement therapy. Patients who need renal support are associated with high mortality rates, exceeding 60% in developing countries [3].

Blood urea nitrogen (BUN) and serum creatinine, two current indicators of nephrotoxicity, are neither sensitive nor specific enough, often leading to delayed diagnosis and treatment. As a result, there is a growing demand for new biomarkers that can detect kidney damage at earlier stages, thus enabling timely intervention [4]. Biomarkers indicate the presence of a disease or damage due to toxic exposure, offering insight into the mechanism of injury. Promising candidates have been identified, with urine-based markers being lovely due to the non-invasive nature of collection [5].

One promising biomarker is KIM-1 (Kidney Injury Molecule-1), an adhesion molecule produced in the proximal convoluted tubule (PCT). Urinary concentrations of KIM-1 rise during ischemia or drug toxicity, often with early detection seen in response to cisplatin, gentamicin, and cyclosporine toxicity. In some cases, an increase in KIM-1 levels is detected within 48 h of exposure to nephrotoxic agents, well before a drop in GFR [3]. Another key biomarker, beta-2 microglobulin, is a protein that lymphocytes manufacture and is a marker of tubular injury, especially in inflammatory conditions. Studies on kidney transplantation have shown its usefulness in detecting calcineurin inhibitor toxicity [5].

Clusterin, a protein involved in cellular stress responses, has demonstrated greater accuracy in diagnosing tubular damage than creatinine, especially in patients treated with nephrotoxic drugs like cisplatin, vancomycin, and gentamicin [3]. Its early rise, similar to KIM-1, suggests its potential for earlier detection of tubular damage. Cystatin C, another promising biomarker, is freely filtered by the kidneys and reabsorbed in the proximal tubules. It has shown a better correlation with renal toxicity from drugs like amphotericin B, vancomycin, and polymyxin when compared to creatinine, particularly in patients with cirrhosis or other conditions affecting stable kidney function [6].

In addition to biomarkers, proteinuria is a key indicator of kidney damage. Under normal conditions, high molecular weight proteins are restricted from migrating through the glomerulus into the nephron. Still, in pathological states, proteins like albumin, transferrin, and immunoglobulin G can be detected in urine. These proteins are early markers of glomerular damage, often linked to conditions like diabetes and immune disorders [3]. Low molecular weight proteins, such as β2-microglobulin and α1-microglobulin, are also filtered and reabsorbed in the kidney. An increase in their urinary concentration can signal tubular damage or overload, making proteinuria a valuable tool for detecting nephrotoxicity early [6].

This study aims to evaluate the role of proteinuria and serum cystatin C for the early detection of nephrotoxicity in acutely poisoned patients admitted to Sohag University Hospitals. It seeks to determine the efficacy of these markers in assessing the severity of kidney injury due to toxic exposure. Additionally, it investigates the differences in the elevation of cystatin C and proteinuria levels caused by nephrotoxic drugs compared to non-nephrotoxic medications, helping to better understand their diagnostic value in distinguishing the underlying cause of renal damage.

## Methods

This prospective study was carried out between April 2021 and the end of August 2021 on one hundred individuals who had been acutely poisoned by medications and poisons that had a nephrotoxic impact directly or indirectly. Before taking part, all patients or their family members were asked to sign an informed consent form and were given the choice to accept or decline.

### Subjects

**Fig. 1:**
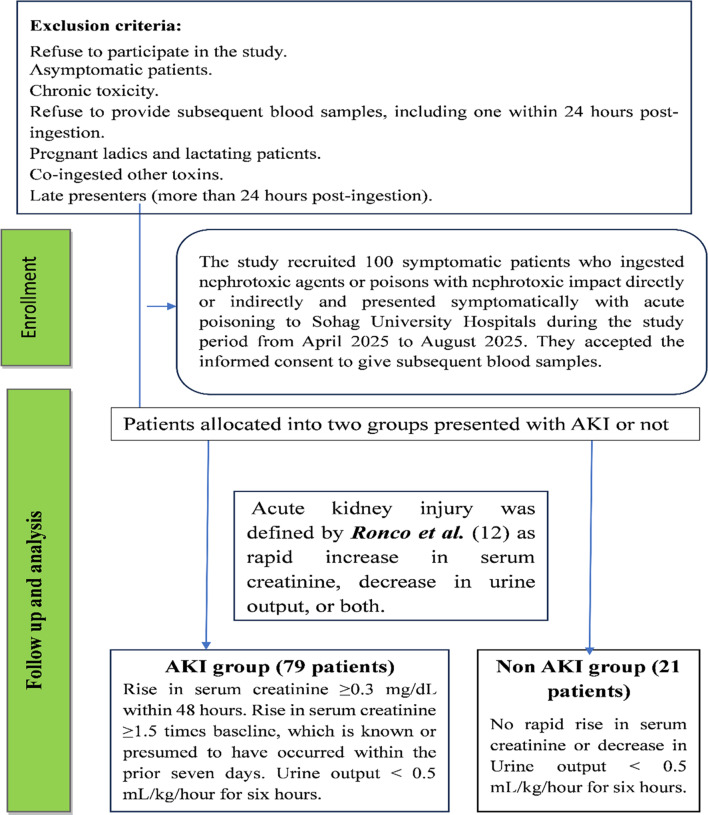
Flow chart of inclusion and exclusion criteria of the patients in the study

The study recruited 100 patients who ingested nephrotoxic agents or poisons with nephrotoxic impact and presented them to Sohag University Hospitals. The inclusion criteria required participants to be symptomatic and provide at least four blood samples, including one within 24 h post-ingestion. Pregnant ladies and lactating patients were excluded, as well as those who had co-ingested other toxins, late presenters (more than 24 h post-ingestion), asymptomatic individuals, and patients who passed away before the first sample collection. Fig. 1.

### Acute kidney injury classification

Acute kidney injury (AKI) was classified using the Acute Kidney Injury Network (AKIN) criteria, similar to other studies exploring AKI biomarkers [7–9]. Since baseline serum creatinine (sCr) measurements from 3 months before renal injury were unavailable for participants, the baseline sCr was estimated as the lowest creatinine value measured during hospitalization or follow-up. This approach has been used in previous studies. Biomarker data were grouped based on post-ingestion time, with maximum biomarker concentrations (Cmax) assessed in 8-hour windows (Cmax 0–8 h, Cmax 8–24 h, Cmax 24–48 h) and for the 0–48 h period post-ingestion [10, 11].

Acute kidney injury was defined by Ronco et al. [12] as the rapid increase in serum creatinine, decrease in urine output, or both.

### Biomarker assays

All samples were assayed in duplicate using standard operating procedures. Serum and urinary creatinine were measured using the Jaffe method on a Hitachi 912 automated analyzer. Serum albumin was assessed using a Mindray semiautomated analyzer. Serum cystatin C was analyzed using microparticle-enhanced immunoturbidometry on a KonelabTM clinical chemistry analyzer.

The study involved comprehensive data collection from 100 acutely poisoned patients at Sohag University Hospitals. Patients’ sociodemographic information included age, sex, residence, and occupation. A detailed history was obtained, focusing on the type of toxic agent, co-ingestions, time between intoxication and hospital admission, route of exposure, mode of intoxication (homicidal, suicidal, or accidental), and presenting symptoms across various systems, including gastrointestinal, cardiovascular, respiratory, genitourinary, and nervous systems. Past medical history, including psychiatric and other medical conditions, was also documented.

Clinical examinations assess vital signs, complexion, skin condition, pupil response, and specific local examinations, such as chest, cardiac, abdominal, and neurological assessments. Investigations included serum electrolyte levels, kidney function tests, blood gas analysis, and biomarker assays to classify acute kidney injury using the AKIN criteria. Additional tests, like abdominal ultrasound imaging, were performed to evaluate kidney status, and data were gathered for statistical analysis.

Treatment measures varied based on patient needs and included emergency interventions (e.g., intubation, oxygen administration), supportive care (e.g., intravenous fluids, antibiotics), and decontamination methods (e.g., emesis, activated charcoal). Enhanced elimination techniques, such as dialysis, were utilized as necessary. Outcomes were documented, noting the admission site, duration of hospital stay, and patient disposition, including recovery status and mortality rates.

Upon admission, a questionnaire was completed to capture each patient’s demographic characteristics, including gender, age, type and amount of medication or toxin consumed, and the time elapsed between toxin ingestion and hospital admission. Additionally, 5 ml venous blood and urine samples were collected from patients in the emergency department or ICU for further analysis.

Statistical analysis involved descriptive statistics, Mann-Whitney and chi-square tests for group comparisons, correlation analyses, linear regression for AKI severity predictors, logistic regression for factors influencing nephrotoxicity, and Receiver Operating Characteristic (ROC) curve analysis for diagnostic accuracy, with *p* < 0.05 considered significant.

Ethical approval was obtained from the Medical Research Ethics Committee of the Faculty of Medicine at Sohag University. On 15/09/2020, the ethical committee approval was obtained for Soh-Med-20-09-15. Data were anonymously collected in the study. The Medical Research Ethics Committee of Sohag Faculty of Medicine, Sohag University, approved the study after written informed consent was obtained from all patients or their first-degree relatives, if appropriate. All of the study’s procedures followed the applicable ethical standards, guidelines, and legislation outlined in the Helsinki Declaration and its subsequent amendments.

## Results

In the current study, the age distribution among the patients ranged from 2 to 58 years with a mean ± SD of 34.98 ± 12.95 years. There were 72 (72%) males and 28 (28%) females. There were 10 (10%) students, 1 (1%) teacher, 67 (67%) workers, 2 (2%) housewives and 20 (20%) unemployed. The majority of the studied patients were from rural areas (82%) while urban just (18%) (Table 1).

**Table 1:** Percentage of sociodemographic distribution of the studied patients according to age, sex, residence, marital State, and occupation

Sociodemographic variables			
Age (years)			
Mean ± SD	34.98 ± 12.95		
Range	2–58		
Sex	Number		Percentage (%)
Male	72		72%
Female	28		28%
Total	100		100%
Occupation			
Student	10		10%
Teacher	1		1%
Worker	67		67%
Housewife	2		2%
Unemployed	20		20%
Total	100		100%
Marital state			
Single	50		50%
Married	49		49%
Widow	1		1%
Total	100		100%
Residence			
Urban	18		18
Rural	82		82
Total	100		100%

According to the nephrotoxic drug cause, 13 (13%) were admitted to ICU due to paraphenylenediamine administration, followed by 11 (11%) patients due to opiate administration and 8 (8%) patients due to organophosphorus poisoning (Table 2).

**Table 2:** Percentage of nephrotoxic drugs or toxins among the studied patients

Cause of toxicity	Number	Percentage (%)
Acetaminophen	1	1%
Allopurinol	1	1%
Aluminium phosphide	4	4%
Aminoglycosides	3	3%
Amphetamine	1	1%
Antidepressant	1	1%
Antihistaminic	1	1%
Aspirin	2	2%
Benzodiazepines	1	1%
Carbamate	3	3%
Carbamazepine	1	1%
Clozapine	2	2%
Carbon monoxide toxicity	2	2%
Contrast	2	2%
Corrosive	3	3%
Declophen	1	1%
Digoxin	1	1%
Dormex	3	3%
Fluoxetine	1	1%
Haloperidol	1	1%
Hashish	5	5%
Ibuprofen	1	1%
Lithium	1	1%
Metformin	2	2%
Methanol	7	7%
Non-steroidal anti-inflammatory drugs	1	1%
Opiate	11	11%
Organophosphorus poisoning	8	8%
Potassium bromide	3	3%
Paraphenylenediamine	13	13%
Snake Bite	2	2%
Statins	1	1%
Theophylline	3	3%
Tramadol	6	6%
Warfarin	1	1%
Total	100	100%

On the 1st day, serum creatinine ranged from 0.77 to 5.03 mg/dL (mean ± SD: 1.67 ± 0.6 mg/dL), and on the 2nd day, it ranged from 0.3 to 8 mg/dL (mean ± SD: 2.98 ± 1.35 mg/dL). Serum urea levels varied from 17 to 190 mg/dL (mean ± SD: 75.45 ± 37.57 mg/dL), potassium ranged from 2.2 to 7.7 mEq/L (mean ± SD: 4.39 ± 1.26 mEq/L), sodium ranged from 124 to 173 mEq/L (mean ± SD: 138.51 ± 7.48 mEq/L), and urine output ranged from 50 to 3000 ml/day (Table 3).

**Table 3:** Serum creatinine at 1st, 2nd day (mg/dL), Urea, potassium level, sodium level, as well as UOP in the studied patients

Serum creatinine at 1st day (mg/dL)	
Mean ± SD	1.67 ± 0.6
Range	0.77–5.03
Serum creatinine at 2nd day (mg/dL)	
Mean ± SD	2.98 ± 1.35
Range	0.3–8
Serum urea (mg/dL)	
Mean ± SD	75.45 ± 37.57
Range	17–190
Potassium level (mEq/L)	
Mean ± SD	4.39 ± 1.26
Range	2.2–7.7
Sodium level (mEq/L)	
Mean ± SD	138.51 ± 7.48
Range	124–173
Urine output (ml/day)	
Mean ± SD	1179.5 ± 737.8
Range	50–3000

We found a significant relationship between acute renal toxicity and Serum creatinine on the 1st and 2nd day (mg/dL) as well as sodium level. Still, there was no statistical significance between acute renal toxicity and blood urea, potassium level, and UOP (Table 4).

**Table 4:** Mann Witney test analysis of serum creatinine on 1st and 2nd day (mg/dL), Urea, potassium level, sodium level, as well as UOP in the two groups of the studied patients

Parameters		Acute renal toxicity		*P* value
		Yes 79(79%)	No 21(21%)	
Urine Output	1200(300,1600)	1200(800,1900)	1200(300,1600)	0.48
Creatinine day 1	1.4(1.27,0.1.8)	1.8(1.4,2.4)	1.4(1.27,0.1.8)	0.026
Creatinine day 2	3(2.5,3.7)	1.7(1.4,2.0)	3(2.5,3.7)	0.000
Urea	73(54,106)	61(35,86.5)	73(54,106)	0.166
Potassium	4.2(3.5,5.2)	4(3.4, 5)	4.2(3.5,5.2)	0.54
Sodium	139(135,142)	136(132,140)	139(135,142)	0.050

In this study, 70% of patients were admitted to the ICU, 10% to the intermediate care unit, and 20% as inpatients. The median ICU stay was 4 days (IQR: 3–7 days). Regarding the overall hospital stay at Sohag University Hospitals, 39% were admitted for 2 days, 31% for 1 day, 21% for 3 days, 4% for 4–5 days, and 5% for 10–25 days. Decontamination via gastric lavage was performed in 13% of patients, while 3% underwent emesis. Dialysis was required for 18% of patients.

Supportive treatments included fluid administration in 85% of cases, mechanical ventilation in 50%, and antiemetic therapy in 23%. In terms of outcomes, 57% of patients achieved complete recovery, 2% had partial recovery, 2% were discharged against medical advice, 8% developed chronic kidney disease (CKD), and 31% of patients died. There was no statistically significant difference among nephrotoxic agents in their association with acute kidney injury (AKI), although paraphenylenediamine (PPD) accounted for the highest percentage of cases leading to AKI, then opiate and organophosphorus (Table 5).

**Table 5:** Comparison between acute kidney injury group and non-acute kidney injury regarding the cause of toxicity

Toxin involved	Acute kidney injury		Total	P-value by chi-square
	No	Yes		
Acetaminophen	1 (4.8%)	0 (0%)	1 (1%)	0.2
Allopurinol	1 (4.8%)	0 (0%)	1 (1%)	
Aluminum phosphide	0 (0%)	4 (5.1%)	4 (4%)	
Aminoglycosides	2 (9.5%)	1 (1.3%)	3 (3%)	
Amphetamine	0 (0%)	1 (1.3%)	1 (1%)	
Antidepressant	0 (0%)	1 (1.3%)	1 (1%)	
Antihistaminic	0 (0%)	1 (1.3%)	1 (1%)	
Asprin	2 (9.5%)	0 (0%)	2 (2%)	
Benzodiazepines	1 (4.8%)	0 (0%)	1 (1%)	
Carbamate	0 (0%)	3 (3.8%)	3 (3%)	
Carbamazepine	1 (4.8%)	0 (0%)	1 (1%)	
Clozapine	0 (0%)	2 (2.5%)	2 (2%)	
Carbon monoxide toxicity	1 (4.8%)	1 (1.3%)	2 (2%)	
Contrast	1 (4.8%)	1 (1.3%)	2 (2%)	
Corrosive	1 (4.8%)	2 (2.5%)	3 (3%)	
Declophen	0 (0%)	1 (1.3%)	1 (1%)	
Digoxin	0 (0%)	1 (1.3%)	1 (1%)	
Dormex	0 (0%)	3 (3.8%)	3 (3%)	
Fluoxetine	0 (0%)	1 (1.3%)	1 (1%)	
Haloperidol	0 (0%)	1 (1.3%)	1 (1%)	
Hashish	1 (4.8%)	4 (5.1%)	5 (5%)	
Ibuprofen	0 (0%)	1 (1.3%)	1 (1%)	
Lithium	0 (0%)	1 (1.3%)	1 (1%)	
Metformin	0 (0%)	2 (2.5%)	2 (2%)	
Methanol	3 (14.3%)	4 (5.1%)	7 (7%)	
NSAID	0 (0%)	1 (1.3%)	1 (1%)	
Opiate	2 (9.5%)	9 (11.4%)	11 (11%)	
Organophosphorus poisoning	1 (4.8%)	7 (8.9%)	8 (8%)	
Potassium bromide	1 (4.8%)	2 (2.5%)	3 (3%)	
Paraphenylenediamine	1 (4.8%)	12 (15.2%)	13 (13%)	
Snake Bite	0 (0%)	2 (2.5%)	2 (2%)	
Statins	0 (0%)	1 (1.3%)	1 (1%)	
Theophylline	0 (0%)	3 (3.8%)	3 (3%)	
Tramadol	1 (4.8%)	5 (6.3%)	6 (6%)	
Warfarin	0 (0%)	1 (1.3%)	1 (1%)	
Total	21 (100%)	79 (100%)	100 (100%)	

Linear regression analysis was used to assess predictors of acute renal toxicity, and significant associations were found with the following variables: serum creatinine on day 1 (*P* = 0.000), serum creatinine on day 2 (*P* = 0.000), proteinuria ACR (*P* = 0.023), and cystatin C (*P* = 0.000). Therefore, these parameters can be considered reliable predictors of acute kidney injury (AKI) (Table 6).

**Table 6:** The linear regression analysis of the numeric study tools as predictors of acute renal toxicity

Coefficients					
	Unstandardized coefficients		Standardized coefficients	T	Sig.
	B	Std. Error	Beta		
(Constant)	0.579	0.545		1.062	0.291
UOP	-5.420E-6	0.000	− 0.010	− 0.137	0.891
Delay per hour	− 0.001	0.001	− 0.062	− 0.833	0.407
Serum creatinine day 1	− 0.195	0.051	− 0.290	-3.816	0.0
Serum urea	0.001	0.001	0.089	1.153	0.252
Serum Creatinine Day 2	0.121	0.024	0.404	5.121	0.0
Potassium level	0.021	0.023	0.065	0.894	0.374
Sodium level	0.005	0.004	0.098	1.350	0.181
Proteinuria ACR	0.000	0.000	0.169	2.318	0.023
Cystatin C	0.113	0.021	0.401	5.313	0.00

**Fig. 2:**
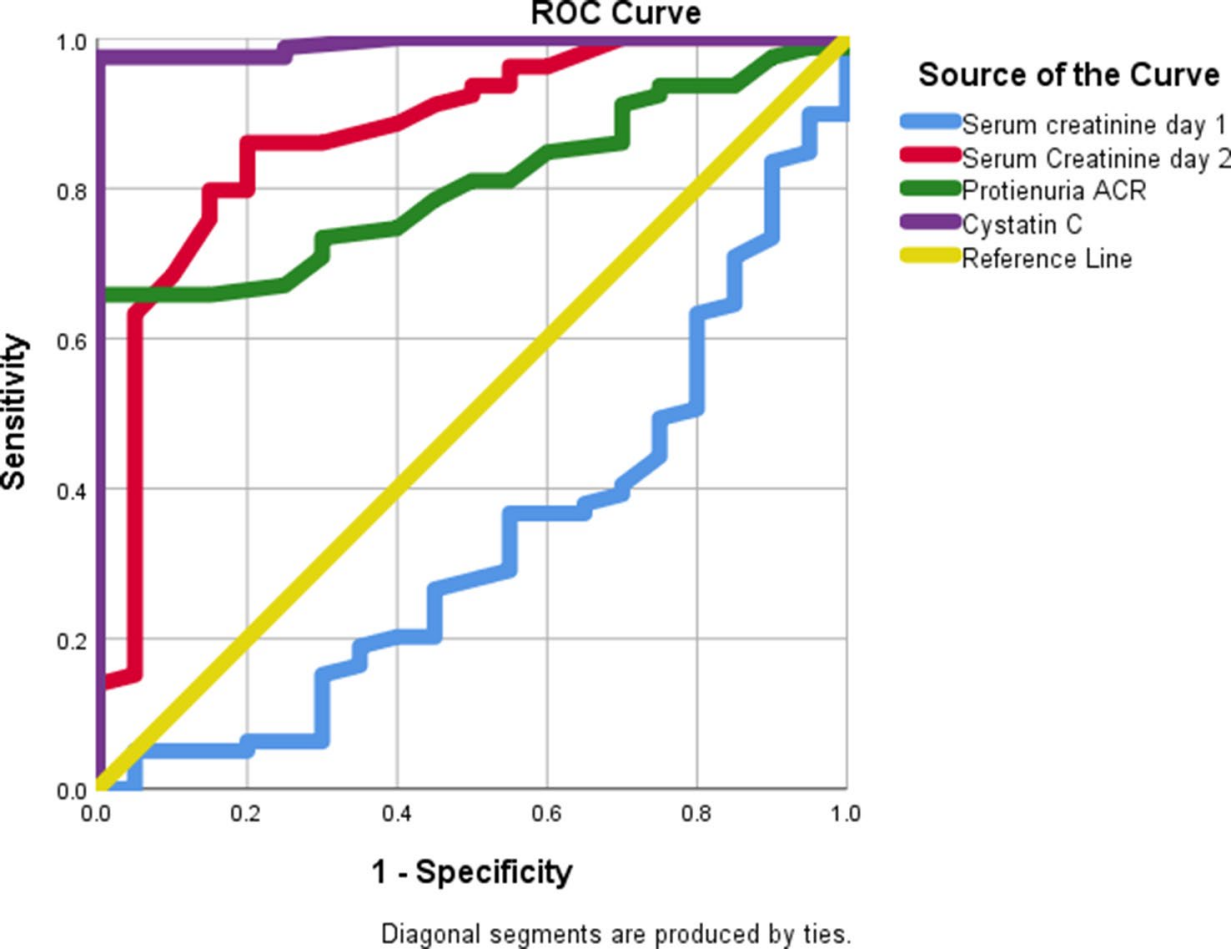
ROC curve of cystatin C, Creatinine Day 1, Creatinine Day 2, and Proteinuria (ACR) as early assessment of acute renal toxicity

**Fig. 3:**
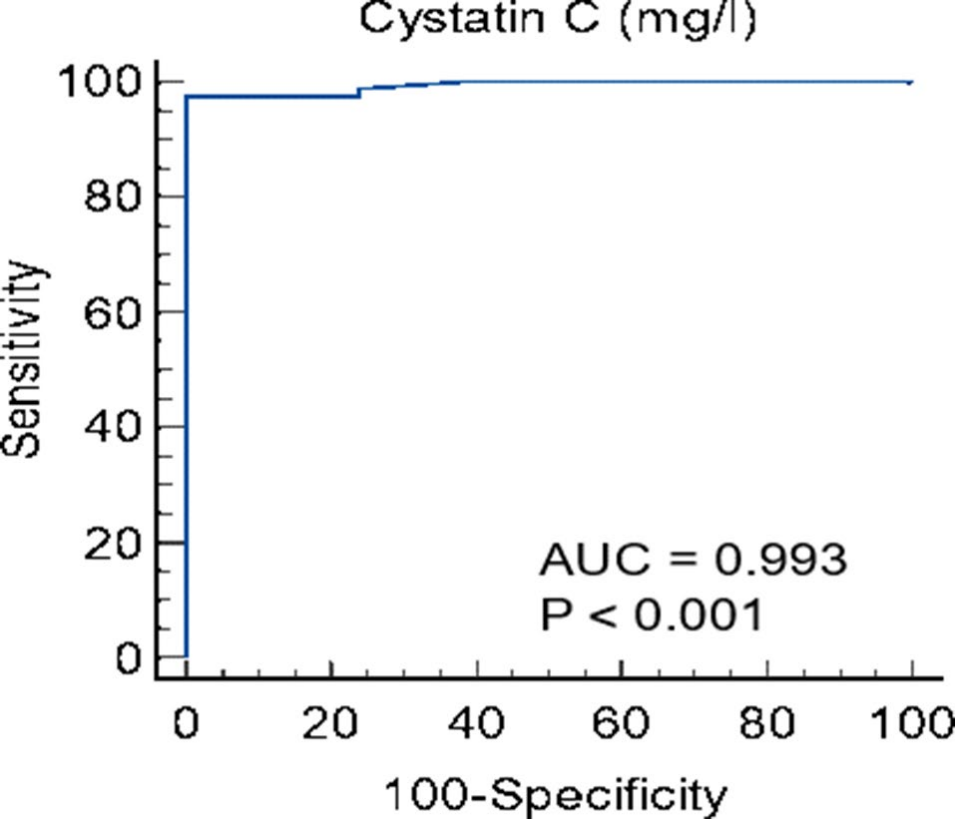
ROC curve of cystatin C as early assessment of acute renal toxicity

**Fig. 4:**
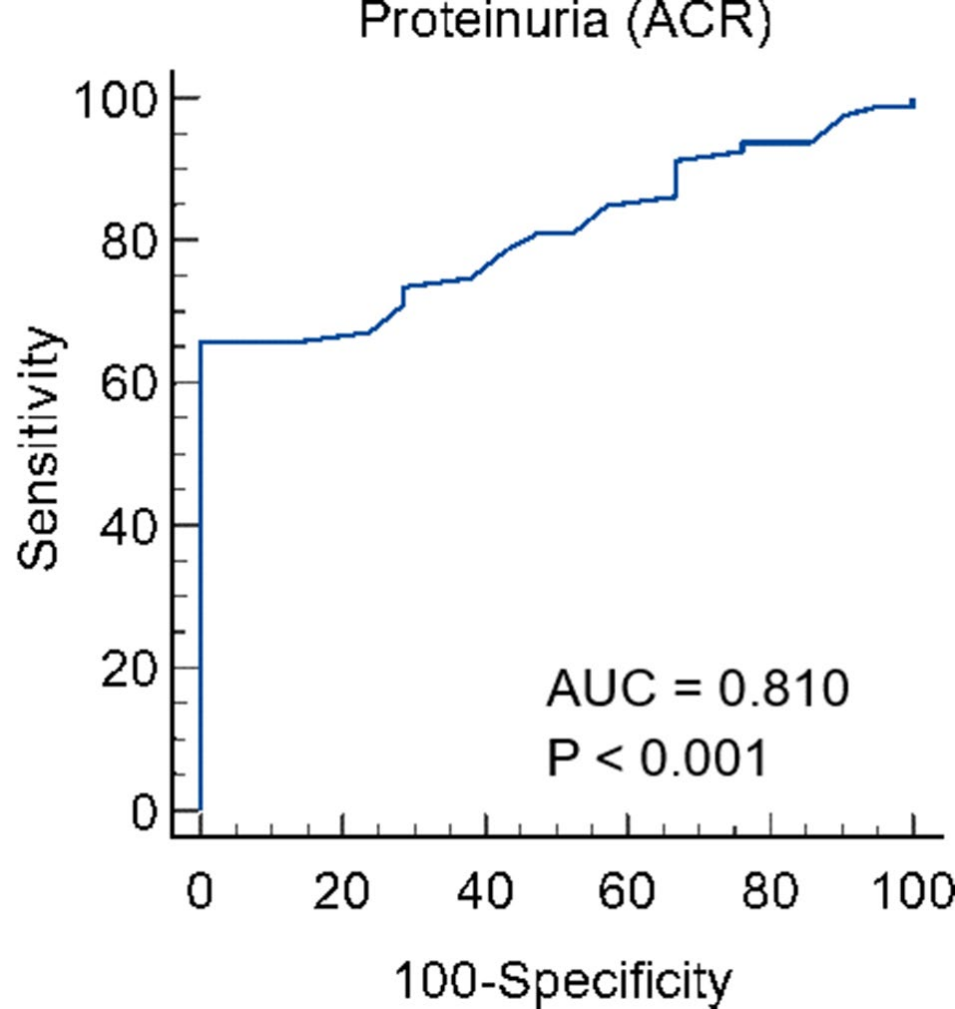
The receiver operating characteristic (ROC) curve of proteinuria (ACR) as an early assessment of acute renal toxicity

Receiver Operating Characteristic (ROC) curve analysis was conducted to evaluate the predictors of acute renal toxicity (see Table 7; Figs. 2 and 3, and 4). The results indicated that cystatin C is a significant predictor of acute renal toxicity, with an area under the curve (AUC) of 0.993 (*P* < 0.001) at a cut-off of > 1.1 mg/L, demonstrating 97.47% sensitivity, 100% specificity, 100% positive predictive value (PPV), and 91.3% negative predictive value (NPV). Similarly, proteinuria (ACR) significantly predicted acute renal toxicity with an AUC of 0.805 (*P* < 0.001) at a cut-off of > 28, achieving 65.82% sensitivity, 100% specificity, 100% PPV, and 43.7% NPV. Additionally, serum creatinine on day 2 was a significant predictor, with an AUC of 0.873 (*P* < 0.001) at a cut-off of > 2.3, resulting in 79.7% sensitivity, 85% specificity, 95.5% PPV, and 53% NPV.

**Table 7:** The diagnostic accuracy, sensitivity, specificity, and accuracy rate of predictors of outcome of creatinine day1 and 2, Cystatin C and proteinuria (ACR) in early assessment of acute renal toxicity

	Cut-off	AUC	Sensitivity	Specificity	PPV	NPV	*P* value
Creatinine 1 mg/dl	> 2.5	0.327	5.1%	95%	66.7%	20.2%	**0.017**
Creatinine2 mg/dl	> 2.3	0.873	79.7%	85%	95.5%	53%	< 0.001*
Cystatin C (mg/l)	> 1.1	0.993	97.47	100	100	91.3	< 0.001*
Proteinuria (ACR)	> 28	0.805	65.82	100	100	43.7	< 0.001*

ACR: albumin creatinine ratio, AUC: area under the curve, PPV: positive predictive value, NPV: negative predictive value, *: significant as P value ≤ 0.05

## Discussion

In the current study, the leading causes of ICU admissions among patients were as follows: Paraphenylenediamine (PPD) administration (13 patients, 13%), opiate administration (11 patients, 11%), and organophosphorus poisoning (8 patients, 8%). This distribution of nephrotoxic drug percentages is similar to findings by Khalil et al. [7], which identified opiates, organophosphorus compounds, methanol, PPD, hashish, and tramadol as common toxins linked to acute kidney injury (AKI).

In contrast, a study by Sweni et al. [8] in South India reported snake bites as the primary cause of AKI, while dichromate, indigenous remedies, and PPD were among the least frequent causes. Notably, no patients with organophosphate or medication overdoses developed AKI.

Similarly, Naqvi [9] in Pakistan identified PPD, methanol, organophosphorus compounds, paraquat, copper sulfate, tartaric acid, phenobarbital, and benzodiazepine toxicity as common AKI causes. Sivakumar and Karthikeyan [10] in India also found snake bites, paraquat, rat poison, copper sulfate, ethylene glycol, and organophosphorus poisoning to be prevalent causes of AKI. These discrepancies highlight the variability in AKI causes across studies, likely influenced by factors such as patient demographics, the potency and composition of snake venom, and differences in healthcare resources [11].

In the current study, the median delay time for patients was 7.5 h, ranging from 4 to 24 h. The modes of toxication included accidental addiction (28 patients, 28%), unintentional non-addiction (20 patients, 20%), criminal acts (4 patients, 4%), iatrogenic causes (11 patients, 11%), and suicidal intentions (37 patients, 37%). No significant correlation was found between delay time and the presence or absence of acute renal toxicity.

Conversely, Khalil et al. [7] reported that most patients were admitted within a delay time of 1–6 h, followed by 6–24 h, and then 1–3 days. Their findings indicated that accidental addiction accounted for 37.14% of cases, followed by suicidal (32.86%), unintentional non-addiction (27.14%), and both iatrogenic and criminal cases (1.43% each). In agreement with these findings, Mostafa et al. [13] concluded that self-poisoning was the predominant mode of poisoning, followed by accidental poisoning and drug overdoses or abuse.

In the current study, 77 patients (77%) ingested the drug or toxin orally. In comparison, 2 patients (2%) inhaled it, 17 patients (17%) received it intravenously, 1 patient (1%) intramuscularly, 2 patients (2%) through bites, and 1 patient (1%) via the dermal route. No significant statistical differences were found regarding the mode of poisoning or route of intake. Khalil et al. [7] also noted that oral exposure was the most common route, consistent with Mostafa et al. [13], who reported similar results among adolescents at Ain Shams University Hospitals. This prevalence may be explained by the ease of use and availability of poisons that can be used orally [14].

In the current study, urinary system manifestations included anuria in 9 patients (9%), oliguria in 15 patients (15%), hematuria in 2 patients (2%), and dark urine in 17 patients (17%). In contrast, Khalil et al. [7] reported that 75.71% of patients exhibited no urinary manifestations. Among those with symptoms, 8.57% had anuria, 8.57% had oliguria, and 7.14% had hematuria. Conversely, Sweni et al. [8] identified oliguria as the most common symptom of renal dysfunction, with similar onset times for oliguria. Naqvi [9] found that 93% of patients experienced oligoanuria, while 86% presented with hematuria.

In the current study, there was a significant relationship between acute renal toxicity and serum creatinine levels on both the 1st and 2nd day (mg/dL) and sodium levels. However, no statistical significance was found between acute renal toxicity and blood urea, potassium levels, or urine output. Khalil et al. [7] reported significant differences in serum creatinine among acutely intoxicated patients across various RIFLE stages, along with substantial variations in serum urea and estimated glomerular filtration rate (eGFR) related to different RIFLE stages.

Sivakumar and Karthikeyan [10] noted that the mean peak creatinine value among 50 patients was 5.13 mg/dL. In contrast, Sweni et al. [8] found mean blood creatinine levels of 6.1 and 6.8 mg/dL, with mean blood urea values of 276 and 318 mg/dL in cases of envenomation and chemical poisoning, respectively.

The current study’s median Cystatin C level was 2.12 mg/L, with an interquartile range (IQR) of 1.21 to 3.28 mg/L. The median proteinuria (albumin-to-creatinine ratio, ACR) was 32, with an IQR of 18.75 to 125.25. A highly significant relationship was observed between acute renal toxicity and both Cystatin C and ACR.

Abdelsalam et al. [15] studied 132 patients (74 males and 58 females), finding that 35 cases (26.5%) developed acute kidney injury (AKI) with increased serum creatinine, according to KDIGO criteria (2012). Their analysis of urinary biomarkers revealed significant increases in urinary KIM-1, Cystatin C, and NGAL in the AKI group on the day of diagnosis and one day before the rise in serum creatinine.

In the current study, 13 patients (13%) underwent gastric lavage for decontamination, while 3 patients were treated with emesis. Dialysis was performed in 18 patients (18%). In contrast, Khalil et al. [7] reported that dialysis was administered to only 21.43% of patients, whereas Sivakumar and Karthikeyan [10] found that dialysis was needed in approximately 86% of patients.

In this study, opiates were linked to the highest percentage of complications or deaths, with a statistically significant difference in outcomes among nephrotoxic agents. However, there was no significant difference in their association with AKI, though paraphenylenediamine caused the most AKI cases. Abdelsalam et al. [14] found that 26.5% of 132 patients developed AKI. However, no significant difference in biomarker levels was observed across groups treated with different platinum-based drugs (PBD) despite cisplatin being the most nephrotoxic. Khalil et al. [7] reported 51.43% complete recovery, 42.86% mortality, and 5.71% chronic kidney disease, while Naqvi [9] noted 72.28% recovery and 20% mortality.

Linear regression analysis in the current study showed that serum creatinine on day 1 and day 2, proteinuria ACR, and cystatin C were significantly associated with AKI, making them valuable predictors.

Dieterle et al. [16] found urinary cystatin C was better than BUN and SCr for detecting glomerular injury, while clusterin and urinary protein were better for tubular injury. Serum cystatin C was superior to urinary cystatin C for AKI detection [17], and combining plasma cystatin C with NGAL improved early AKI diagnosis and severity assessment [18].

In the current study, cystatin C effectively predicted acute renal toxicity (AUC = 0.993, *P* < 0.001). Similarly, Abdelsalam et al. [15] found cystatin C highly effective for the early detection of nephrotoxicity from platinum-based drugs (AUC = 1, *P* < 0.001). In contrast, Lin et al. [19] reported that NGAL increased significantly 12 h after cisplatin in AKI cases, while urinary cystatin C was a poor marker for cisplatin-induced AKI. This discrepancy may be due to differences in sample size, AKI definitions, and cystatin C measurement methods.

## Conclusion

Proteinuria and cystatin C are reliable predictors for the early detection of nephrotoxicity in acutely poisoned patients. Furthermore, these biomarkers are effective in indicating and assessing the severity of kidney injury resulting from toxicity.

## Data Availability

Availability of data and materialsData are available upon reasonable request from the corresponding author.

## References

[1] Kane-Gill SL, Goldstein SL. Drug-induced acute kidney injury: a focus on risk assessment for prevention. Crit Care Clin. 2015;31(4):675–84. 26410137 10.1016/j.ccc.2015.06.005

[2] Perazella MA. Pharmacology behind common drug nephrotoxicities. Clin J Am Soc Nephrol. 2018;13(12):1897–908. 29622670 10.2215/CJN.00150118 PMC6302342

[3] Caires RA, Silva VT, Burdmann E, Coelho FO, Costalonga EC. Drug-induced acute kidney injury. In: Ronco C, Bellomo R, Kellum JA, Ricci Z, editors. Critical care nephrology. 3rd ed. Philadelphia: Elsevier; 2019:214–21.

[4] Awdishu L, Mehta RL. The 6R’s of drug-induced nephrotoxicity. BMC Nephrol. 2017;18(1):124. 28372552 10.1186/s12882-017-0536-3 PMC5379580

[5] Griffin BR, Faubel S, Edelstein CL. Biomarkers of drug-induced kidney toxicity. Ther Drug Monit. 2019;41(2):213–26. 30883514 10.1097/FTD.0000000000000589 PMC6436396

[6] Balci C, Uzun Q, Arici M, Hayran SA, Yüce D, Ünal S. Nephrotoxicity of piperacillin-tazobactam combined with Vancomycin: should it be a concern? Int J Antimicrob Agents. 2018;52(2):180–4. 29649586 10.1016/j.ijantimicag.2018.03.024

[7] Khalil M, Radwan RA, Moussa ME, Mohamed S. Evaluation of acute kidney injuries among acutely intoxicated patients by RIFLE classification. Mansoura J Forensic Med Clin Toxicol. 2022;30(1):45–57.

[8] Sweni S, Meenakshisundaram R, Sakthirajan R, Rajendiran C, Thirumalaikolundusubramanian P. Acute renal failure in acute poisoning: prospective study from a tertiary care centre of South India. J Ren Care. 2012;38(1):22–8. 21951386 10.1111/j.1755-6686.2011.00255.x

[9] Naqvi R. Acute kidney injury from different poisonous substances. World J Nephrol. 2017;6(3):162. 28540206 10.5527/wjn.v6.i3.162 PMC5424438

[10] SivaKumar DK, Karthikeyan M. Study of clinical profile and outcome of acute kidney injury in acute poisoning and envenomation. Int J Adv Med. 2018;5(2):249–56.

[11] Paul J, Dasgupta S. Early prediction of acute kidney injury by clinical features of snakebite patients at the time of hospital admission. North Am J Med Sci. 2012;4(5):216. 10.4103/1947-2714.95903 PMC3359432 22655280

[12] Ronco C, Bellomo R, Kellum JA. Acute kidney injury. Lancet. 2019;394(10212):1949–64. 31777389 10.1016/S0140-6736(19)32563-2

[13] Mostafa H, Rezk N, Khater A, Abdelaziz S. Pattern and severity of acute poisoning among adolescents: A six months prospective study in poison control Center-Ain Shams university hospitals. Ain Shams J Forensic Med Clin Toxicol. 2014;23(2):160–72.

[14] Tarvadi PV, Bakkannavar SM, Manjunath S, Palimar V, Kumar GP, Shetty M. Trends of poisoning among children at Kasturba hospital, manipal. J Health Allied Sci NU. 2013;3(02):025–8.

[15] Abdelsalam M, Elmorsy E, Abdelwahab H, Algohary O, Naguib M, El Wahab AA, Eldeeb A, Eltoraby E, Abdelsalam A, Sabry A, El-Metwally M. Urinary biomarkers for early detection of platinum based drugs induced nephrotoxicity. BMC Nephrol. 2018;19:1–8. 30180818 10.1186/s12882-018-1022-2 PMC6123931

[16] Dieterle F, Perentes E, Cordier A, et al. Urinary clusterin, Cystatin C, b2- microglobulin, and total protein as markers to detect drug-induced kidney injury. Nat Biotechnol. 2010;28:463–9. 20458316 10.1038/nbt.1622

[17] Soto K, Coelho S, Rodrigues B, et al. Cystatin C as a marker of acute kidney injury in the emergency department. Clin J Am Soc Nephrol. 2010;5:1745–54. 20576828 10.2215/CJN.00690110 PMC2974372

[18] Basu RK, Wong HR, Krawczeski CD, Wheeler DS, Manning PB, Chawla LS, Devarajan P, Goldstein SL. Combining functional and tubular damage biomarkers improves diagnostic precision for acute kidney injury after cardiac surgery. J Am Coll Cardiol. 2014;64(25):2753–62. 25541128 10.1016/j.jacc.2014.09.066 PMC4310455

[19] Lin HY, Lee SC, Lin SF, Hsiao HH, Liu YC, Yang WC, Hwang DY, Hung CC, Chen HC, Guh JY. Urinary neutrophil gelatinase-associated Lipocalin levels predict cisplatin-induced acute kidney injury better than albuminuria or urinary Cystatin C levels. Kaohsiung J Med Sci. 2013;29(6):304–11. 23684135 10.1016/j.kjms.2012.10.004 PMC11916332

